# Nutritional analysis of vegetable soybean [*Glycine max* (L.) Merrill] accessions in Eastern India

**DOI:** 10.3389/fnut.2025.1643470

**Published:** 2025-09-04

**Authors:** Devireddy Meghana, Kodihalli Somashekar Rachana, Rabi Sankar Pan, Sandeep Kumar, Sujit Kumar Bishi, Danish Jawed, Mahesh Kumar Mahatma, Meenu Kumari, Reshma Shinde, Brij Bihari Sharma, Akshay Talukdar, Prakash Kumar, Ankit Kumar Sinha, Ramakrishnan Madhavan Nair, Anil Dahuja, Anup Das

**Affiliations:** ^1^ICAR-Research Complex for Eastern Region, Farming System Research Centre for Hill and Plateau Region, Ranchi, Jharkhand, India; ^2^Division of Vegetable Science, ICAR-Indian Agricultural Research Institute, New Delhi, Delhi, India; ^3^Autiomation and Plant Engineering Division, ICAR-National Institute of Secondary Agriculture, Ranchi, Jharkhand, India; ^4^ICAR-Indian Institute of Agricultural Biotechnology, Ranchi, Jharkhand, India; ^5^ICAR-National Research Centre on Seed Spices, Ajmer, Rajasthan, India; ^6^Department of Vegetable Science, Rani Lakshmi Bai Central Agricultural University - Jhansi, Jhansi, Uttar Pradesh, India; ^7^Division of Genetics and Plant Breeding, ICAR-Indian Agricultural Research Institute, New Delhi, Delhi, India; ^8^Division of Statistical Genetics, ICAR-Indian Agricultural Statistics Research Institute, New Delhi, Delhi, India; ^9^World Vegetable Center South Asia, ICRISAT Campus, Hyderabad, Telangana, India; ^10^Division of Biochemistry, ICAR-Indian Agricultural Research Institute, New Delhi, Delhi, India

**Keywords:** vegetable soybean, isoflavones, tocopherols, trypsin inhibitors, fatty acids

## Abstract

**Introduction:**

Vegetable soybean (*Glycine max* L. Merrill), commonly consumed as edamame, represents a nutritionally dense crop with growing global demand. Despite its potential to address dietary diversification and enhance functional food markets, systematic evaluation of its biochemical traits remains limited, particularly in Eastern India where malnutrition and low crop diversity persist.

**Methods:**

Thirty-four vegetable soybean genotypes were assessed under field conditions using a randomized block design with three replications. Quantitative analysis of proteins, carbohydrates, sugars, oil, fatty acids, vitamins, isoflavones, tocopherols, phenolics, trypsin inhibitor activity, and lipoxygenase activity was conducted using HPLC, GC, and spectrophotometric assays. Multivariate analyses including PCA, correlation, and hierarchical clustering were performed to identify trait associations and superior genotypes.

**Results:**

Significant differences (*p* ≤ 0.05) were observed among genotypes for all the parameters. Protein content varied between 25.7 and 34.9%, while sugars ranged from 13.5 to 24.6%. Our analysis showed that vegetable soybean genotypes, particularly AGS-190 and AGS-456, contained oleic acid levels exceeding 50%, which is considerably higher than those typically reported in grain soybean, thereby indicating improved oxidative stability. Isoflavone content was more than 14 mg/100 g in several accessions, whereas AGS-292 and AGS-610 recorded lower trypsin inhibitor and lipoxygenase activities, respectively, improving consumer suitability. Considerable diversity was also observed for tocopherols, phenolics, and vitamin C, indicating scope for enhancing antioxidant potential. Multivariate analyses identified a set of superior lines integrating multiple desirable traits, marking them as promising candidates for biofortification, functional food development, and breeding of next-generation vegetable soybean cultivars.

**Discussion:**

The findings align with earlier studies reporting wide genotypic variation in protein, oil, sugar content and bioactive compounds of vegetable soybeans. However, unlike grain soybeans, the vegetable soybean genotypes showed higher oleic acid and lower linoleic acid, likely due to differences in harvest stage and seed maturity. Certain parameters showed ranges, comparatively lower than earlier studies, likely due to genotype-specific differences and agro-climatic conditions. These variations emphasize the need for region-specific screening to identify nutritionally rich and consumer-preferred cultivars.

## 1 Introduction

The most recent Child Malnutrition Estimates for 2023, jointly issued by UNICEF, the World Health Organization (WHO), and the World Bank Group, underscore the fact that the highest numbers of malnourished children are concentrated in sub-Saharan Africa and South Asia (Joint Child Malnutrition Estimates−2023). Between 2019 and 2021 in India, the prevalence of stunting and underweight among children exceeded over 30%, with wasting affecting over 20% children. A substantial proportion of these cases originated in Eastern India. In adults, the underweight prevelance exceeded 20% in both genders, and overweight was observed in more than 30% adults (1). Heavy dependence on the three primary crops (wheat, maize, and rice) and poor dietary habits significantly contribute to malnutrition (2). Soybeans stand out as a globally significant crop, serving as both a pulse and oilseed crop due to their nutritional and functional qualities. Soybean protein is an economical source of high-quality dietary protein, containing all essential amino acids, including lysine, which is often deficient in other staple crops. Soy protein also has a relatively high biological value and a protein digestibility-corrected amino acid score (3). Furthermore, soybean oil is valued for its high polyunsaturated and low saturated fatty acid composition, improving cardiovascular health. Soybeans also harbor functional compounds such as isoflavones, which have been associated with reduced incidences of various cancers, cardiovascular diseases, and post-menopausal symptoms (4, 5).

**Figure 1:**
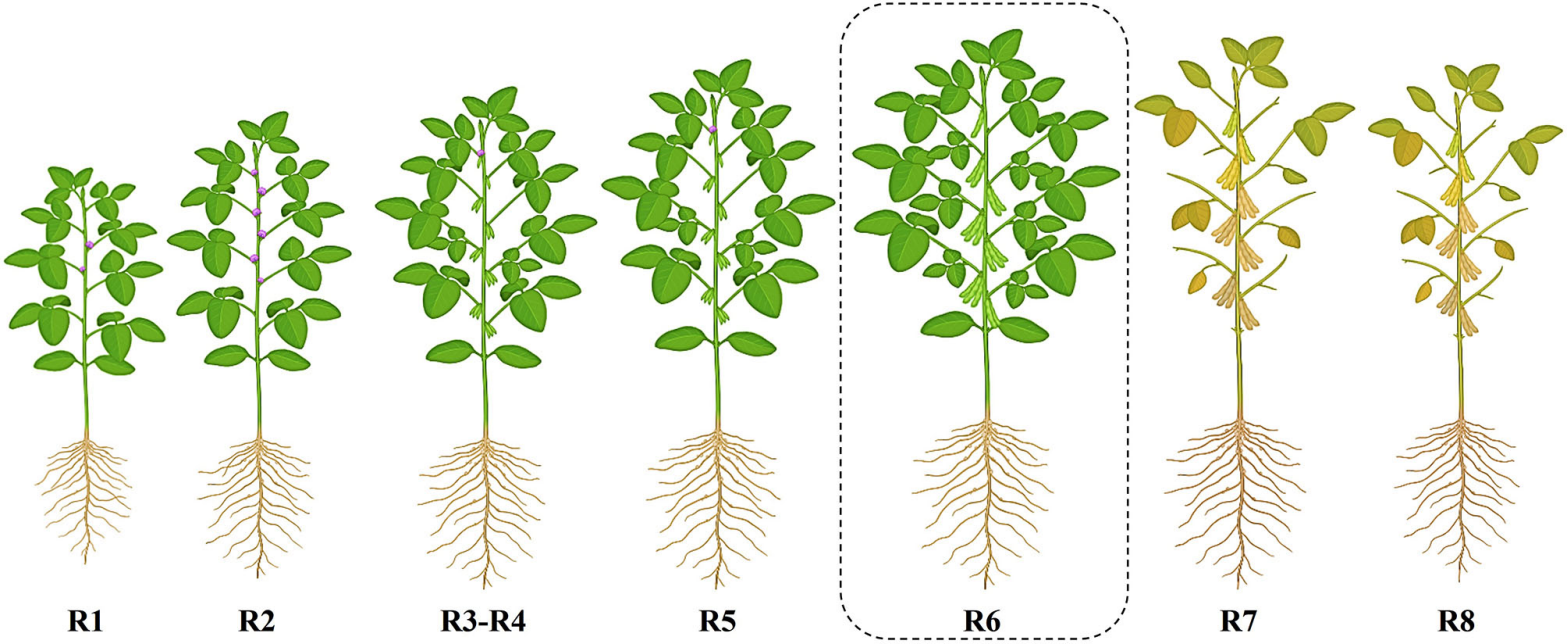
Overview of the reproductive (R) stage progression in vegetable soybean, where R1—of flowering; R2—full bloom; R3 and R4—pod development stages; R5—beginning of seed; R6—full seed; R7—beginning of maturity; R8—full maturity.

Unlike grain soybeans, which are typically harvested at full maturity (R8 stage) when the seeds are yellow and dry, vegetable soybeans are harvested earlier. They are picked at the R6 stage, where the pods are still tender and green and contain ~80% of their seeds, before they reach full maturity (Figure 1) (6). Vegetable soybeans can be distinguished from grain soybeans by several key characteristics: they have larger seed sizes, typically weighing over 60–75 g per 100 seeds at fresh weight, possess a sweet taste, and lack the typical beany flavor associated with grain soybeans. The tender green pods of vegetable soybeans are highly valued in culinary contexts, as they can be utilized in diverse ways, including as snacks, additions to salads, or as ingredients in stir-fried dishes (7). In terms of protein content and quality, vegetable soybeans are regarded as a superior protein source compared to vegetable pigeon peas and green peas (8). Furthermore, vegetable soybeans exhibit higher levels of vitamins, notably vitamin A, compared to grain soybeans (9). Additionally, it contains higher amounts of essential minerals such as calcium, potassium, phosphorus, magnesium, iron, copper, zinc, and manganese in comparison to green peas.

The Eastern Plateau and Hill Region (EPHR), encompassing parts of Chhattisgarh, Jharkhand, Madhya Pradesh, Maharashtra, Odisha, and West Bengal, falls within the agro-climatic zone VII of India. Here, the temperature ranges from 10 to 27 °C, and the annual rainfall varies between 80 and 150 cm. The region's predominant soil types are red and yellow, occasionally interspersed with laterite and alluvial soils. Water resources are limited due to the plateau's structure and intermittent streams, leading to a reliance on rainfed agriculture practices. Vegetable soybeans have gained prominence in these regions due to its successful cultivation in rainfed uplands during kharif, and their nutritional richness addresses the malnutrition challenges in these areas. Additionally, it plays a role in diversifying crops, particularly because many other legumes are primarily available during the rabi season. Being a leguminous crop, it also aids in nitrogen fixation in the atmosphere, contributing to soil replenishment.

Nonetheless, vegetable soybean production accounts for < 2% of global soybean production, and its nutritional profile has not received as much attention as mature soybean (10). It holds substantial promise for the food industry, can aid in biofortification studies, and ultimately contributes to combating malnutrition. Hence, the assessment of genotypes for their nutritional and anti-nutritional contents is crucial, as it enables the identification of genotypes that possess higher nutritional value and lower levels of antinutritional components. This knowledge is invaluable for addressing nutritional deficiency problems. Identifying genotypes with superior nutritional profiles can help mitigate potentially adverse effects on human health and improve the overall acceptability of the crop. To address this gap, an analysis of vegetable soybean's biochemical composition, including isoflavones, tocopherols, trypsin inhibitor (TI) activity, oil content, and fatty acid composition of the genotypes maintained in the Eastern Plateau and Hill Region, was carried out in the present study. Moreover, analysis of proteins, carbohydrates, sugars, and micronutrients was also performed.

## 2 Materials and methods

### 2.1 Experimental material

The experimental materials comprised 34 different genotypes of vegetable soybeans, sourced from various regions with diverse phenotypic and grain characteristics (Supplementary Table S1). All plants were cultivated in a randomized block design with three replications at ICAR-Research Complex for Eastern Region and Farming System Research Center for Hill and Plateau Region, situated in Plandu, Ranchi district, Jharkhand, India. After harvesting at the R6 stage, tender green pods were stored immediately in a deep freezer at −80 °C until further use. For biochemical analysis (except enzymatic analysis), the seeds were deshelled from the pods and dried at 60 °C using a hot air oven. The dried seeds were finely ground into powder and filtered with the help of 100-mesh sieves to attain uniformity. Three replications were used to estimate all traits, and the trait values are represented per 100 g of dry weight of seeds.

The reagents and chemicals used for high-performance liquid chromatography (HPLC) estimation used in this study were of HPLC grade. Isoflavone standards, including daidzin, glycitin, genistin, daidzein, glycitein, and genistein, were sourced from Merck (Darmstadt, Germany). Ethanol, methanol, sodium hydroxide, tannic acid, gallic acid, sulfuric acid, and other chemicals required for the experiments were purchased from HiMedia (Mumbai, India). Additional reference standards, such as α, β, γ, and δ tocopherols, as well as solvents such as methanol, acetonitrile, and water were obtained from Sigma-Aldrich (St. Louis, MO, USA). For the sample preparation, centrifuge tubes from Tarsons (Kolkata, India) and sample vials with screw caps from Shimadzu (Kyoto, Japan) were used. Sample centrifugation was performed with a Centrifuge 5810 R from Eppendorf (Hamburg, Germany), and absorbance measurements were carried out using Evolution™ One Plus from ThermoFisher Scientific (Waltham, Massachusetts, United States).

### 2.2 Total carbohydrates, total sugars, and reducing sugars (%)

The total carbohydrates and total sugars were estimated by the Anthrone method with slight modifications (11). A measure of 100 mg of powdered seed sample was extracted with 10 ml of 80% ethanol, and the contents were centrifuged for 15 min at 4,000 rpm. The supernatant was collected and the volume adjusted to 10 ml. A measure of 0.1 ml of this extract was mixed with 4 ml of anthrone reagent and kept in a boiling water bath for 10 min. After cooling down, the intensity of the blue-green colored complex formed was measured spectrophotometrically at 630 nm.

A standard glucose stock solution was prepared by dissolving 0.1 g of glucose in 100 ml of distilled water. From this, a working standard was made by diluting 10 ml of the stock solution to 100 ml of distilled water. To prepare the standard series, 0, 0.2, 0.4, 0.6, 0.8, and 1.0 ml of the working standard were transferred into separate test tubes. Distilled water was added to bring the total volume in each tube to 1 ml, yielding final glucose concentrations of 0, 20, 40, 60, 80, and 100 ppm, respectively, with 0 ppm serving as the blank. Each tube then received 4 ml of anthrone reagent. The tubes were heated in a boiling water bath for 8 min and then rapidly cooled to room temperature. The absorbance of each solution was measured at 630 nm using a UV-visible spectrophotometer. A standard curve was plotted with glucose concentration on the *X*-axis and the corresponding absorbance on the *Y*-axis. This curve was then used to determine the carbohydrate concentration in test samples by comparing their absorbance values. The carbohydrate content in the samples was calculated using the following formula.


$$\begin{array}{l} \text{Carbohydrate content }\left(\%\right)=\\ \;\frac{\text{concentration of glucose from graph }\times \text{100}}{\text{The weight of test the sample}} \end{array}$$

### 2.3 Crude oil (%) content and fatty acid profiling

The crude oil was extracted using a Soxhlet apparatus. A volume of 5 g of finely powdered seed samples were weighed (*W*_1_), placed into the thimble, and plugged with fat-free cotton wool. The thimble was placed in a Soxhlet apparatus that was connected to a pre-weighed beaker for oil collection. The oil was extracted with the solvent *n*-hexane for 6–7 h continuously. After cooling, the oil was collected from the flask, and the content was recorded. The defatted sample was taken out and kept for drying, and the weight of the dried sample was recorded (*W*_2_).

The crude oil percentage was calculated using the formula:


$$\begin{array}{l} \text{Crude oil }\left(\text{\%}\right)=\frac{\begin{array}{c} \text{Initial weight of sample }\left({\text{W}}_{1}\right)\left(g\right)\\ -\text{ weight of oven dried sample }\left({\text{W}}_{2}\right)\left(g\right)\times \;100 \end{array}}{\text{Initial weight of the sample }\left({\text{W}}_{1}\right)\;\left(g\right)} \end{array}$$

For fatty acid profiling, the fatty acids were converted into fatty acid methyl esters (FAMEs) through transesterification, and these FAMEs were analyzed using the gas chromatography system (Agilent make 7890A) according to the method given by Chaudhari et al. (12) with minor modifications to the temperature program. After extraction, 100 μl of oil was mixed with 5 ml of *n*-hexane. To this mixture, 200 μl of potassium methoxide was added, vortexed, and kept aside at room temperature for 30 min. A volume of 1 μl of FAME was injected into the injection port at a split ratio of 1:50. FAMEs were separated on an HP5 capillary column (30 m × 320 μm × 0.25 μm), with a flow of 0.5 ml carrier gas. Oven temperature was set at 170 °C and then gradually increased to 205 °C at the rate of 5 °C/min, then to 211 °C at the rate 1 °C/min, and finally to 255 °C at a rate of 10 °C/min and detected using a flame ionization detector. Detector temperature was kept at 280°C. Fatty acids were identified based on the retention time of standard fatty acids (FAME mixture RM3, Merck) and expressed as the percentage area of individual fatty acids.

### 2.4 Total protein content (%)

Total nitrogen in the seed samples was estimated using the Kjeldahl method (13) with some modifications. To 0.5 g of powdered, defatted sample, 5 ml of the digestion mixture was added along with a blank. To the mixture, 10 ml of concentrated H_2_SO_4_ was added, and the sample was added to the Kjeldahl digestion unit. When the color changed from sky blue to green, the samples were removed from the chamber. A measure of 20 ml of distilled water was added and mixed properly. The samples were fed into a distillation set. The extract from the distillation set was titrated against 0.1 N H_2_SO_4_ for nitrogen estimation.


$$\text{Total nitrogen }(\%)=\frac{\text{Titer value }\times \text{ 0}.\text{1 N }{\text{H}}_{2}{\text{SO}}_{\text{4}}\text{ factor }\times \text{100}}{\text{Weight of the plant sample}}$$

The total nitrogen content was multiplied by the conversion factor of 6.25 to calculate total protein.

### 2.5 Vitamin C

Vitamin C content of vegetable soybean seed was determined using the 2,6-dichloro phenol-indophenol visual titration method (14). A volume of 5 g of fresh green seed samples were weighed and crushed with 10 ml of 4% oxalic acid, filtered, and made up to 100 ml. A measure of 5 ml of extract was added to a conical flask containing 10 ml of 4% oxalic acid solution and titrated against dye until a pink color appeared. The ascorbic acid content was calculated using a formula.


$$\begin{array}{l} \text{Ascorbic acid }\left(\frac{\text{mg}}{\text{100 g}}\right)=\\ \;\frac{\text{0}.\text{5 }\times \text{ Titrate value}\times \text{ volume made up }\times \text{ 100}}{\text{Aliquot taken for estimation }\times \text{ weight of the sample}} \end{array}$$

### 2.6 Estimation of tocopherols

Tocopherols (mg/100 g) were estimated using HPLC, where a C_18_ silica column was used as the stationary phase (15, 16). The soybean oil extracted using n-hexane was dissolved in 5 ml of methanol and passed through a 0.5-μm filter to get a clear filtrate. A measure of 20 μl of the filtrate obtained from the samples was injected into HPLC and ran for 20 min. Separation was accomplished by 100% methanol at a flow rate of 1.0 ml/min for 20 min. Oven temperature was maintained at 40 °C. Detection was carried out using a photodiode array (PDA) detector with a wavelength of 295 nm. The amount of each tocopherol (α, β+γ, and δ) present in the sample was estimated from the peak area.

### 2.7 Total phenolic (TP) content

Total phenolic content was determined using the Folin–Ciocalteu reagent (FCR) following the method described by Singleton et al. (17). A volume of 1 g of sample was homogenized with 20 ml of 80% methanol three times, filtered, and made up to a volume of 100 ml. A measure of 0.5 ml of aliquot of the extract was taken, and 0.2 ml of 1N FCR was added, followed by 3.3 ml of distilled water. After 2 min, 1 ml of Na_2_CO_3_ (20%) solution was added, and the reaction mixture was incubated for 30 min at room temperature before measuring at 765 nm.

### 2.8 Estimation of isoflavones

The extraction of the sample was conducted based on the principle of alkaline hydrolysis, converting isoflavones to their aglycone forms (18). A measure of 500 mg of ground soybean seed powder was mixed with a 1:1 acetonitrile–water solution. After homogenization and sonication, samples were centrifuged at 5,000 rpm for 15 min. at 4 °C. The supernatant was collected and mixed with KOH, incubated for 3 h, neutralized with KH_2_PO_4_, and dried. Finally, the dried samples were reconstituted with 40% DMSO and subjected to high-performance liquid chromatography (HPLC). The separation was performed by a C_18_ column (2.1 mm × 50 mm) using acetonitrile (v/v 0.1% formic acid) and water (v/v 0.1% formic acid) as solvents A and B, respectively, at 40 °C. A measure of 5 μl of the prepared sample extract was injected into the column inlet. The samples were run using the following time program: (% solvent A/%solvent B): 0 min (10/90), 5 min (30/70), 20 min (10/90), and 25 min (10/90). Detection was carried out using a photodiode array PDA detector with a wavelength of 254 nm. The amount of individual isoflavones present in the sample was estimated using the peak area of the sample in the equation derived from the standard graphs.

### 2.9 Lipoxygenase enzyme activity

Lipoxygenase (LOX) enzyme activity of vegetable soybean seeds was estimated using the method given by Dahuja et al. (19). A measure of 100 mg defatted flour was weighed and homogenized with 1 ml of cold phosphate buffer (0.1 M, pH = 6.5). The mixture was centrifuged at 12,000 g for 30 min at 4 °C. The supernatant was collected and used for the LOX activity assay. A measure of 50 μl of this enzyme extract was added to 2.95 ml of the linoleic substrate solution, and an increase in absorbance was recorded at 234 nm for 5 min at 25 °C. The LOX enzyme activity was expressed in ΔA/min/mg soluble protein, where ΔA = change in absorbance per min.

### 2.10 Estimation of trypsin inhibitors

Trypsin inhibitors were estimated using THE ISO method 14902:2001 and The AOCS method (20) with slight modifications. A volume of 1 g of seed powder was extracted with 50 ml of 10 mM NaOH in a beaker, using a magnetic stirrer for 3 h. Samples were diluted so that 2 ml of the sample extract could inhibit 30%−70% of the trypsin used as a standard. Substrate was added to the sample, and later, trypsin was added; after 10 min, the reaction was terminated using acetic acid. The entire process was carried out in a hot water bath. Absorbance was taken using a UV-visible spectrophotometer at 410 nm and expressed in trypsin inhibitor units (TIUs)/mg, representing the reduction in trypsin activity (where one trypsin unit is equal to a 0.01 absorbance increase).

### 2.11 Statistical analysis

Data collected from triplicate measurements were analyzed for analysis of variance (ANOVA) using the “PROC GLM” procedure in SAS 9.4. To identify significant differences between means, Tukey's HSD test was applied at a significance level of *P*-value of < 0.05. Correlation analysis utilized the “corrplot” and “Hmisc” packages in R version 4.0.4, allowing for detailed visualization of relationships between variables. Cluster analysis was an essential part of the study, performed with the “cluster” package in R. For visualizing complex data matrices, the “heatmap.2” function from the “gplots” package was used to create comprehensive heatmaps. Principal component analysis (PCA) was conducted with the “FactoMineR” and “factoextra” packages in R, producing biplots that effectively depicted variable relationships. Desirable genotypes containing a high amount of isoflavones, α-tocopherols, oil content, and unsaturated fatty acids were identified using formula 1, whereas genotypes containing low trypsin inhibitor activity and saturated fatty acids were identified using formula 2.


$$\begin{array}{l} \text{Formula }1:\;{\text{X}}_{\text{i}}>\;{\text{X}}_{\text{p}}+\;{\text{sd}}^{1}\;+\;{\text{sd}}^{2}\\ \text{Formula }2:\;{\text{X}}_{\text{i}}<\;{\text{X}}_{\text{p}}-\;{\text{sd}}^{1}\;-\;{\text{sd}}^{2}, \end{array}$$

where X_i_ = mean of individual genotype;

X_p_ = population mean;

sd^1^ = standard deviation of individual genotype; and

sd^2^ = standard deviation of population.

## 3 Results and discussion

### 3.1 Variation in major nutritional factors

#### 3.1.1 Total carbohydrates (%), total, and reducing sugars

Total carbohydrates and total sugars are important parameters, where sugars tend to increase consumer preferences toward any genotype. On the contrary, it could be undesirable where the goal is to produce a crop with a lower sugar content. In the present study, the mean of total sugar content ranged from 13.49 ± 0.4% to 24.57 ± 0.63%, with an average mean value of 19.39% (Table 1). Total sugar content is a key parameter in assessing the nutritional quality of vegetable soybeans (8). The highest sugar content was observed in AGS-404 (24.57 ± 0.63%), followed by Swarna Vasundhara (23.66 ± 0.40%). The mean for reducing sugars ranged from 2.10 ± 0.17% for AGS-458 to 5.08 ± 0.34% for Swarna Vasundhara. The differences were statistically significant for both total sugar content and reducing sugars, indicating a strong genotypic effect on both (Table 2). A similar range of total carbohydrates and reduced sugars have been reported in earlier studies on vegetable soybeans (8, 9, 21, 22).

**Table 1:** Mean performance of 34 vegetable soybean genotypes for major nutrients.

**Genotypes**	**Moisture (%)**	**Total carbohydrates (%)**	**Reducing sugars (%)**	**Total sugar (%)**	**Total oil (%)**	**Total protein (%)**
AGS-190	68	25.08 ± 0.09^i − l^	3.79 ± 0.07^b − d^	19.80 ± 0.65^d − i^	18.67 ± 0.65^a^	33.31 ± 1.56^ab^
AGS-292	62	21.70 ± 0.21^p^	2.39 ± 0.22^k − m^	16.73 ± 0.41^k − m^	16.27 ± 0.50^a − g^	31.52 ± 1.12^ab^
AGS-329	64	22.63 ± 0.45^n − p^	3.55 ± 0.26^c − e^	17.50 ± 0.13^i − m^	16.83 ± 1.01^a − e^	30.94 ± 1.40^ab^
AGS-331	65	23.06 ± 0.82^m − o^	2.98 ± 0.35^f − h^	18.55 ± 0.68^g − l^	17.10 ± 0.74^a − e^	31.99 ± 1.57^ab^
AGS-332	66	22.32 ± 0.48^°*p*^	2.92 ± 1.69^f − i^	16.37 ± 0.66^lm^	15.77 ± 0.46^a − g^	27.94 ± 1.19^ab^
AGS-333	68	26.25 ± 0.49^g − l^	3.89 ± 0.03^bc^	20.06 ± 0.43^c − h^	18.40 ± 0.57^a − c^	32.97 ± 1.50^ab^
AGS-334	69	26.49 ± 0.36^g − k^	4.24 ± 0.18^b^	21.70 ± 0.59^b − e^	15.50 ± 0.46^b − g^	34.95 ± 1.32^a^
AGS-336	70	27.61 ± 0.51^e − h^	3.82 ± 0.52^b − d^	20.43 ± 0.45^c − g^	15.67 ± 0.52^a − g^	31.78 ± 1.79^ab^
AGS-337	70	25.33 ± 0.57^i − l^	2.29 ± 0.39^lm^	19.01 ± 0.44^f − k^	14.67 ± 1.00^d − i^	31.33 ± 1.73^ab^
AGS-338	65	22.83 ± 0.48^n − p^	2.51 ± 0.46^i − m^	18.60 ± 0.33^g − l^	14.07 ± 0.54^e − j^	32.63 ± 1.85^ab^
AGS-339	65	20.86 ± 0.38^p^	2.76 ± 0.45^g − k^	17.75 ± 0.40^h − m^	17.26 ± 0.59^a − d^	32.39 ± 2.07^ab^
AGS-357	62	24.40 ± 0.21^l − n^	3.69 ± 0.09^cd^	17.87 ± 0.20^h − m^	16.20 ± 0.18^a − g^	30.53 ± 0.91^ab^
AGS-380	62	22.43 ± 0.59^°*p*^	2.93 ± 0.36^f − i^	16.98 ± 0.54^j − m^	16.57 ± 0.24^a − f^	26.71 ± 0.62^b^
AGS-402	71	24.67 ± 0.29^k − m^	2.46 ± 0.02^j − m^	19.63 ± 0.20^d − i^	14.93 ± 0.23^d − h^	29.72 ± 1.06^ab^
AGS-404	77	29.55 ± 0.18^b − d^	3.54 ± 0.32^c − e^	24.57 ± 0.63^a^	15.37 ± 0.55^c − g^	33.48 ± 1.40^ab^
AGS-406	66	22.14 ± 0.26^°*p*^	2.20 ± 0.45^m^	13.49 ± 0.41^n^	16.47 ± 0.20^a − f^	27.96 ± 1.21^ab^
AGS-447	63	22.65 ± 0.40^n − p^	2.30 ± 0.10^lm^	15.69 ± 1.01^mn^	15.67 ± 0.38^a − g^	32.93 ± 1.29^ab^
AGS-456	62	28.57 ± 0.58^c − f^	3.70 ± 0.53^cd^	18.14 ± 0.64^g − m^	18.60 ± 0.43^ab^	28.67 ± 1.20^ab^
AGS-457	62	24.72 ± 0.30^k − m^	3.25 ± 0.47^ef^	16.92 ± 0.42^j − m^	17.50 ± 0.45^a − d^	29.34 ± 0.92^ab^
AGS-458	64	32.74 ± 0.17^a^	5.00 ± 0.03^a^	21.23 ± 0.40^b − f^	16.40 ± 0.66^a − f^	27.80 ± 1.47^ab^
AGS-459	72	27.99 ± 0.28^d − g^	2.98 ± 0.18^f − h^	19.57 ± 0.35^d − i^	14.50 ± 0.51^d − j^	32.32 ± 1.12^ab^
AGS-460	72	25.30 ± 0.28^i − l^	2.49 ± 0.42^i − m^	19.06 ± 0.46^f − k^	12.30 ± 0.59^h − k^	27.98 ± 1.45^ab^
AGS-461	67	26.91 ± 0.25^f − i^	3.96 ± 0.27^bc^	21.98 ± 0.34^−d^	13.67 ± 0.57^f − j^	32.91 ± 1.16^ab^
AGS-610	63	26.73 ± 0.36^g − j^	3.98 ± 0.42^bc^	19.41 ± 0.36^e − j^	11.77 ± 0.95^i − k^	25.75 ± 1.09^b^
DSB-15	66	25.47 ± 0.27^i − l^	3.14 ± 0.06^e − g^	19.45 ± 0.29^e − j^	13.27 ± 0.96^g − k^	31.57 ± 1.30^ab^
EC595818	67	22.58 ± 0.24^n − p^	2.66 ± 0.08^h − l^	17.52 ± 0.19^i − m^	13.43 ± 0.35^f − k^	32.66 ± 1.39^ab^
EC595823	62	30.69 ± 0.17^b^	3.88 ± 0.10^bc^	21.37 ± 12.34^b − f^	11.60 ± 0.06^jk^	32.19 ± 0.98^ab^
EC595824	68	29.25 ± 0.48^b − e^	4.23 ± 0.03^b^	22.34 ± 0.46^a − c^	15.73 ± 0.69^a − g^	32.92 ± 1.71^ab^
GC-84501-32-1	75	29.85 ± 0.38^bc^	3.41 ± 0.37^de^	22.28 ± 0.37^a − c^	10.34 ± 0.44^k^	29.07 ± 1.79^ab^
Harit Soya	75	29.17 ± 0.30^b − e^	3.71 ± 0.06^cd^	23.08 ± 13.33^ab^	16.57 ± 0.67^a − f^	29.19 ± 1.33^ab^
HAVSB-1	70	25.88 ± 0.15^h − l^	2.10 ± 0.17^m^	18.26 ± 0.54^g − l^	16.33 ± 0.38^a − g^	30.50 ± 1.25^ab^
Karune	70	30.86 ± 0.14^b^	4.82 ± 0.23^a^	22.29 ± 0.25^a − c^	15.50 ± 0.58^b − g^	33.39 ± 1.16^ab^
NRC-105	68	24.85 ± 0.25^j − m^	2.84 ± 0.29^f − j^	18.06 ± 0.28^g − m^	13.65 ± 0.61^f − j^	33.23 ± 1.64^ab^
Swarna Vasundhara	67	30.96 ± 0.24^b^	5.08 ± 0.34^a^	23.66 ± 0.40^ab^	17.47 ± 0.40^a − d^	30.91 ± 0.89^ab^

All presented values are mean of three replications & mean values with different superscript alphabets within the same column are statistically different (*p*-value < 0.05).

**Table 2:** *F*-value and *P*-values for the different biochemical characteristics.

**Biochemical characteristics**	***F*-value**
Carbohydrates	68.14^**^
Reducing Sugars	79.89^**^
Total Sugars	46.53^**^
Total Oil	12.34^**^
Palmitic Acid	5.66^**^
Arachidic Acid	23.35^**^
Behenic Acid	84.42^**^
Lignoceric Acid	7.32^**^
Oleic Acid	628.71^**^
Linoleic Acid	675.75^**^
Linolenic Acid	29.55^**^
Total Protein	2.65^**^
Trypsin Inhibitor Units (TIU)	346.16^**^
Lipoxygenase (LOX)	1,568.48^**^
Vitamin C	1,049.22^**^
Tocopherols	1,641.1^**^
Total Phenols	5,467.66^**^
Isoflavones	33.31^**^

^*^Significant at P ≤ 0.05.

^**^Highly significant at P ≤ 0.005.

#### 3.1.2 Oil content and fatty acid profile

**Figure 2:**
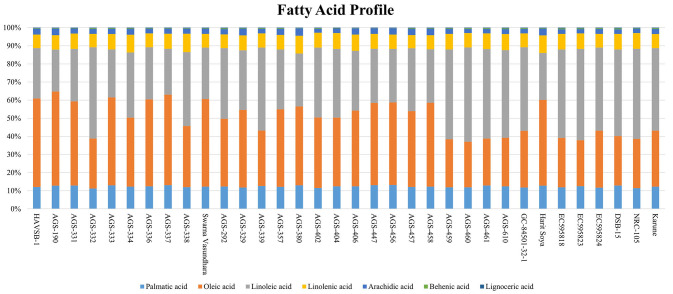
Fatty acid composition of vegetable soybean genotypes.

Total oil content and fatty acid profile are important parameters determining the nutritional quality of vegetable soybeans (7). The total oil content ranged from 10.34 ± 0.44 to 18.67 ± 0.65% (Table 1), with an overall mean of 15.41 ± 0.92%. The maximum content was found in AGS-190 (18.67 ± 0.65%), followed by AGS-456 (18.60 ± 0.43%) and AGS-333 (18.40 ± 0.57%). The range of oil content is similar to that of grain soybeans, as supported elsewhere (21), where no significant difference was found among maturity groups for either protein or oil content. The variation in the fatty acid composition for all 34 vegetable soybean genotypes is displayed in Figure 2 and Supplementary Table S2. Significant genotypic effects were observed for both monounsaturated fatty acids and polyunsaturated fatty acids (PUFAs; Table 2). Oleic acid content varied significantly among all 34 genotypes. The mean value for oleic acid content ranged from 24.96 ± 0.07% to 52.10 ± 0.14%, with an overall mean of 37.75 ± 0.59%. Genotype AGS-190 had the highest oleic acid content of 52.10 ± 0.14%, followed by HAVSB-1, which had 48.92%. The minimum oleic acid content was observed in genotype AGS-460 (24.96 ± 0.07%). Similarly, the mean values of linoleic acid for all the 34 genotypes ranged from 23.16 ± 0.4% to 51.76 ± 0.23% with an overall mean of 37.77 ± 0.59%. Genotype AGS 460 was recorded with the highest linoleic acid content of 51.76 ± 0.23%, followed by AGS-332 (50.55 ± 0.46%). The minimum linoleic acid content was found in AGS-190 (23.16 ± 0.4%). The maximum linolenic acid content was found in genotype AGS-338 (10.13 ± 0.17%), followed by AGS-380 (9.77 ± 0.12%), whereas genotype AGS-457 was found to have a minimum linolenic acid content of 7.24 ± 0.17%. The relatively high content of polyunsaturated fatty acids (PUFAs) accompanied by a high concentration of lipoxygenase contributes to undesirable beany off-flavor in soybeans (23). It is worth noting that the fatty acid profiles in vegetable soybeans differ from those in grain soybeans, as reported by (24, 25), where grain soybeans had lower oleic acid and higher linoleic acid content, affecting the oxidative stability of the extracted oil. This difference could be attributed to factors such as seed size and maturity period, where maturity period showed a negative correlation with oleic acid and a positive one with linoleic and linolenic acids, contributing to higher oil stability in immature seeds. As unsaturation steps successively take place, a harvest at the R6 stage in vegetable soybeans will have better oxidative stability as expected.

#### 3.1.3 Variation in total protein content (%)

Due to the presence of high quantities as well as the quality of protein, soybeans become one of the most promising candidates for alleviating protein energy malnutrition (3). All 34 genotypes under investigation significantly differed in the total protein content of vegetable soybean seeds. The mean values for total protein content ranged from 25.75 ± 1.09% for AGS-610 to 34.95 ± 1.32% for AGS-334, with an overall mean of 30.98% (Tables 1, 2). Protein content is a critical parameter in evaluating the nutritional quality of vegetable soybeans, as it contributes significantly to its value as a plant-based source of essential amino acids for human nutrition. This gives vegetable soybeans an edge against other pulses as the soy protein is known to have a better amino acid composition (26). The selection of cultivars yielding higher total protein content holds promise (27).

### 3.2 Assessment of genotypic differences for undesirable characteristics

#### 3.2.1 Trypsin inhibitor (TI) activity

Trypsin inhibitors constitute a significant class of protease inhibitors present in leguminous seeds. Soybeans are especially rich in such protease inhibitors. The major protease inhibitors present in soybean seeds include a Kunitz inhibitor and Bowman-Birk protease inhibitor (28). The mean value of trypsin inhibitor units ranged from 17.50 ± 0.29 to 39.33 ± 0.17 TIU/mg with an overall mean of 30.84 ± 0.47 TIU/mg (Table 3). Among the 34 genotypes studied, the minimum trypsin inhibitor activity was found in AGS-292 (17.50 ± 0.29 TIU/mg) and the maximum in EC595824 (39.33 ± 0.17 TIU/mg), which are similar to the earlier reported findings, i.e., 43.7 ± 2.93 and 41.0 ± 0.44 (29).

**Table 3:** Mean performance of vegetable soybean genotypes for vitamins, antinutritional factors, and bioactives.

**Genotype**	**LOX enzyme activity (ΔA/min/mg soluble protein × 10^−3^)**	**Tocopherols (mg/100 g)**	**Total phenolic content (mg GAE/100g)**	**Isoflavones (mg/100 g)**	**Vitamin C (mg/100g)**	**Trypsin Inhibitor Units (TIU/mg)**
AGS-190	53.96 ± 0.45^e^	41.78 ± 1.56^p^	1,265.21 ± 2.73^f^	12.70 ± 0.36^cde^	56.42 ± 0.23^g − i^	32.33 ± 0.17^fg^
AGS-292	77.54 ± 0.86^b^	48.64 ± 1.12^mn^	770.00 ± 3.29^k^	11.34 ± 0.85^de^	48.11 ± 0.16^mn^	17.50 ± 0.29^p^
AGS-329	62.87 ± 0.88^c^	83.58 ± 1.40^hi^	1,369.72 ± 4.32^c^	13.29 ± 0.33^cde^	48.27 ± 0.27^mn^	33.00 ± 0.29^fg^
AGS-331	39.52 ± 1.26^ij^	41.91 ± 1.57^°*p*^	723.33 ± 3.33^l^	14.79 ± 0.59^cd^	49.73 ± 0.07^lm^	33.33 ± 0.17^ef^
AGS-332	46.62 ± 0.69^g^	68.26 ± 1.19^k^	1,301.08 ± 2.43^d^	11.73 ± 0.88^de^	57.70 ± 0.18^g^	36.00 ± 0.29^c^
AGS-333	43.28 ± 0.41^h^	34.61 ± 1.50^r^	514.38 ± 6.11^s^	19.47 ± 0.30^ab^	57.41 ± 0.18^gh^	29.67 ± 0.17^j^
AGS-334	39.78 ± 0.65^ij^	56.05 ± 1.32^l^	711.40 ± 5.03^lm^	12.41 ± 0.90^cde^	56.34 ± 0.28^ghi^	37.83 ± 0.17^b^
AGS-336	33.30 ± 0.22^l^	116.44 ± 1.79^de^	892.00 ± 6.81^j^	21.97 ± 0.31^a^	61.37 ± 0.36^f^	34.50 ± 0.29^de^
AGS-337	25.44 ± 0.29^no^	32.84 ± 1.73^r^	1,069.00 ± 2.52^g^	13.95 ± 0.34^cde^	55.37 ± 0.06^ij^	29.50 ± 0.29^j^
AGS-338	26.63 ± 0.46^m − o^	53.39 ± 1.85^l^	605.62 ± 4.21^°*p*^	11.84 ± 0.56^cde^	47.00 ± 0.19^no^	32.50 ± 0.29^fg^
AGS-339	42.76 ± 0.28^h^	81.65 ± 2.07^i^	623.14 ± 2.49°	11.29 ± 0.33^de^	52.38 ± 0.50^k^	37.50 ± 0.29^b^
AGS-357	56.97 ± 0.73^d^	177.41 ± 0.91^a^	593.42 ± 5.12^pq^	11.36 ± 0.33^de^	46.23 ± 0.38°	36.00 ± 0.29^c^
AGS-380	41.70 ± 0.38^hi^	45.38 ± 0.62^no^	1,276.84 ± 4.40^ef^	19.13 ± 0.57^ab^	42.54 ± 0.46^p^	31.67 ± 0.44^gh^
AGS-402	25.17 ± 0.53°	39.60 ± 1.06^pq^	565.06 ± 6.87^r^	18.81 ± 0.52^b^	53.92 ± 0.18^j^	33.00 ± 0.29^fg^
AGS-404	27.54 ± 0.52^mn^	36.96 ± 1.40^qr^	977.97 ± 5.95^h^	12.17 ± 0.58^cde^	72.29 ± 0.36^c^	30.33 ± 0.17^ij^
AGS-406	40.62 ± 0.28^ij^	67.40 ± 1.21^k^	478.82 ± 3.28^t^	22.26 ± 0.58^a^	46.25 ± 0.21°	31.00 ± 0.29^hi^
AGS-447	34.81 ± 0.17^l^	52.54 ± 1.29^lm^	951.71 ± 6.38^i^	13.95 ± 0.35^cde^	38.12 ± 0.13^q^	37.33 ± 0.17^b^
AGS-456	63.64 ± 0.27^c^	76.90 ± 1.20^j^	954.12 ± 1.18^i^	11.53 ± 0.85^de^	33.97 ± 0.05^s^	33.00 ± 0.29^fg^
AGS-457	36.96 ± 0.08^k^	88.97 ± 0.92^g^	888.95 ± 3.43^j^	11.24 ± 0.32^e^	41.54 ± 0.19^p^	30.00 ± 0.29^ij^
AGS-458	33.80 ± 0.24^l^	118.30 ± 1.47^d^	1,260.09 ± 4.13^f^	11.92 ± 0.59^cde^	36.38 ± 0.17^r^	22.50 ± 0.29^n^
AGS-459	26.95 ± 0.09^m − o^	114.00 ± 1.12^e^	1,661.37 ± 4.95^b^	14.24 ± 0.62^cde^	73.52 ± 0.21^bc^	26.50 ± 0.29^l^
AGS-460	28.02 ± 0.09^m^	127.35 ± 1.45^c^	1,819.29 ± 2.30^a^	11.42 ± 0.56^de^	69.64 ± 0.55^d^	27.33 ± 0.17^kl^
AGS-461	45.34 ± 0.06^g^	103.15 ± 1.16^f^	950.00 ± 2.58^i^	11.35 ± 0.90^de^	76.26 ± 0.44^a^	27.67 ± 0.17^kl^
AGS-610	22.07 ± 0.24^p^	119.19 ± 1.09^d^	975.50 ± 4.95^h^	13.67 ± 0.88^cde^	49.55 ± 0.32^lm^	19.83 ± 0.33°
DSB-15	39.12 ± 0.51^j^	72.78 ± 1.30^j^	514.41 ± 2.95^s^	13.18 ± 0.34^cde^	54.83 ± 0.24^ij^	31.83 ± 0.44^gh^
EC595818	27.98 ± 0.36^m^	103.87 ± 1.39^f^	590.71 ± 3.69^pq^	11.85 ± 0.57^cde^	50.28 ± 0.46^l^	28.00 ± 0.29^k^
EC595823	63.86 ± 0.46^c^	113.62 ± 0.98^e^	983.42 ± 1.45^h^	21.97 ± 0.90^a^	46.12 ± 0.05°	28.00 ± 0.29^k^
EC595824	109.58 ± 0.21^a^	142.94 ± 1.71^b^	1,293.23 ± 2.74^de^	15.24 ± 0.59^c^	55.99 ± 0.07^hi^	39.33 ± 0.17^a^
GC-84501-32-1	26.07 ± 0.05^m − o^	46.31 ± 1.79^no^	665.09 ± 8.79^n^	18.76 ± 0.59^b^	69.33 ± 0.87^d^	24.00 ± 0.29^m^
Harit Soya	26.89 ± 0.05^m − o^	76.34 ± 1.33^j^	697.07 ± 8.42^m^	19.82 ± 0.86^ab^	73.60 ± 0.61^bc^	38.17 ± 0.33^ab^
HAVSB-1	32.78 ± 0.29^l^	42.36 ± 1.25^°*p*^	586.5889 ± 3.90^pq^	13.08 ± 0.82^cde^	57.97 ± 0.47^g^	34.83 ± 0.44^cd^
Karune	24.99 ± 0.28°	86.34 ± 1.16^gh^	649.3333 ± 3.28^n^	11.85 ± 0.92^cde^	64.66 ± 0.52^e^	24.00 ± 0.29^m^
NRC-105	51.15 ± 0.28^f^	42.87 ± 1.64^°*p*^	581.9792 ± 6.64^qr^	12.53 ± 0.93^cde^	51.05 ± 0.31^kl^	28.00 ± 0.29^k^
Swarna Vasundhara	49.49 ± 0.53^f^	44.91 ± 0.89^no^	533.7374 ± 3.42^s^	11.12 ± 0.55^e^	74.40 ± 0.31^b^	32.67 ± 0.17^fg^

All presented values are mean of three replications & mean values with different superscript alphabets within the same column are statistically different (*p*-value < 0.05).

#### 3.2.2 Lipoxygenase (LOX) enzyme activity

Soybean oil fraction contains a high content of unsaturated fatty acids. Lipoxygenase acts on unsaturated fatty acids, such as linoleic acid, to generate hexanals, which contribute toward off-flavor, thereby decreasing consumer acceptability (23). Therefore, various breeding in recent years have focused on reducing lipoxygenase activity, including the development of null lines (23, 30). The mean lipoxygenase (LOX) enzyme activity of 34 vegetable soybean genotypes is summarized in Table 3. LOX activity ranged from 22.07 ± 0.24 to 109.58 ± 0.21 ΔA/min/mg soluble protein × 10^−3^, with an overall mean of 41.98 ΔA/min/mg soluble protein × 10^−3^. The lowest LOX activity was observed in AGS-610 (22.07 ± 0.24 ΔA/min/mg soluble protein × 10^−3^), followed by Karune (24.99 ± 0.28 ΔA/min/mg soluble protein × 10^−3^), while the highest activity was recorded in EC595824 (109.58 ± 0.21 ΔA/min/mg soluble protein × 10^−3^). These values are comparable to previously reported values of 8 ± 1.22 and 27.0 ± 2.18 ΔA/min/mg soluble protein × 10^−3^ (31). Due to protein denaturation, lipoxygenase loses almost all its activity at higher processing temperatures, such as blanching/boiling, roasting, and extrusion. Therefore, traditional cooking methods more than suffice to remove LOX activity.

### 3.3 Comparative analysis of vitamins and bioactive compounds

#### 3.3.1 Vitamin C content

Ascorbic acid (vitamin C) is a vital antioxidant important for both plants and humans (32). The mean performance of 34 vegetable soybean genotypes for vitamin C content (mg/100 g) is presented in Table 3. A significant difference was observed among all genotypes (Table 2), with a range of 33.97 ± 0.05% to 76.26 ± 0.44%. The highest vitamin C content was recorded in AGS-461 (76.26 ± 0.44 mg/100 g), followed by Swarna Vasundhara (74.40 ± 0.31 mg/100 g) and Harit Soya (73.60 ± 0.61 mg/100 g). These findings align with previous research (33, 34), which reported mean values ranging from 34.8 to 88.7 mg/100 g seed. Enhanced levels of vitamin C are also desirable, as it aids in the absorption of iron by chelation.

#### 3.3.2 Variation in tocopherols

Tocopherols are the primary antioxidants present in the lipophilic fraction (35). The mean value of the tocopherol content is represented in Table 3. Among 34 genotypes studied, genotype AGS-357 recorded a maximum tocopherol content of 177.41 ± 0.91 mg/100 g, followed by EC595824 (142.94 ± 1.71 mg/100 g). These results are in agreement with previous research (25), in which tocopherol content ranged from 42.2 to 331.1 mg/100 g in soybeans. Similar findings were also previously reported (36) for tocopherol levels in vegetable soybeans.

#### 3.3.3 Total phenolic (TP) content

In soybeans, too, several phenolics are present, such as p-hydroxybenzoic acid, vanillic acid, and ferulic acid (37, 38). Many compounds belonging to the phenolic class are known to have high antioxidant activity. Phenolics also become essential in combating different stresses in plants. The minimum and maximum total phenolic contents were recorded in genotypes AGS-406 [478.82 ± 3.28 mg gallic acid equivalent (GAE)/100 g] and AGS-460 [1,819.29 ± 2.30 mg gallic acid equivalent (GAE)/100 g], respectively, and showed a significant genotypic effect (Tables 2, 3). The values reported in the present study are higher than the earlier findings (36), i.e., 68–139 mg GAE/100 g of seed.

#### 3.3.4 Isoflavones

Isoflavones are a major class of bioactive compounds present in soybeans. They mediate a symbiotic association with rhizobium and can also serve as precursors for glyceollin, a class of phytoalexins. In humans, isoflavones are considered beneficial due to their anticancerous activities (39). The mean values for isoflavone content ranged from 11.12 ± 0.55 to 22.26 ± 0.58 mg/100 g. A significant genotypic effect was observed among the genotypes for isoflavones (Table 2). Interestingly, the minimum isoflavone content was recorded in Swarna Vasundhara (11.12 ± 0.55 mg/100 g), as shown in Table 3. These findings align with previous research conducted in a previous study (33), which reported mean values ranging from 22.67 to 92.62 mg/100 g. Kumar et al. (22) also studied total isoflavone content in vegetable soybeans and found similar mean values in the range of 8.64–33.19 mg/100 g. Moreover, the ability of isoflavones to inhibit lipoxygenase makes them interesting from a breeding perspective (40, 41).

### 3.4 Interrelationship between nutritional, antinutritional factors, and bioactives

**Figure 3:**
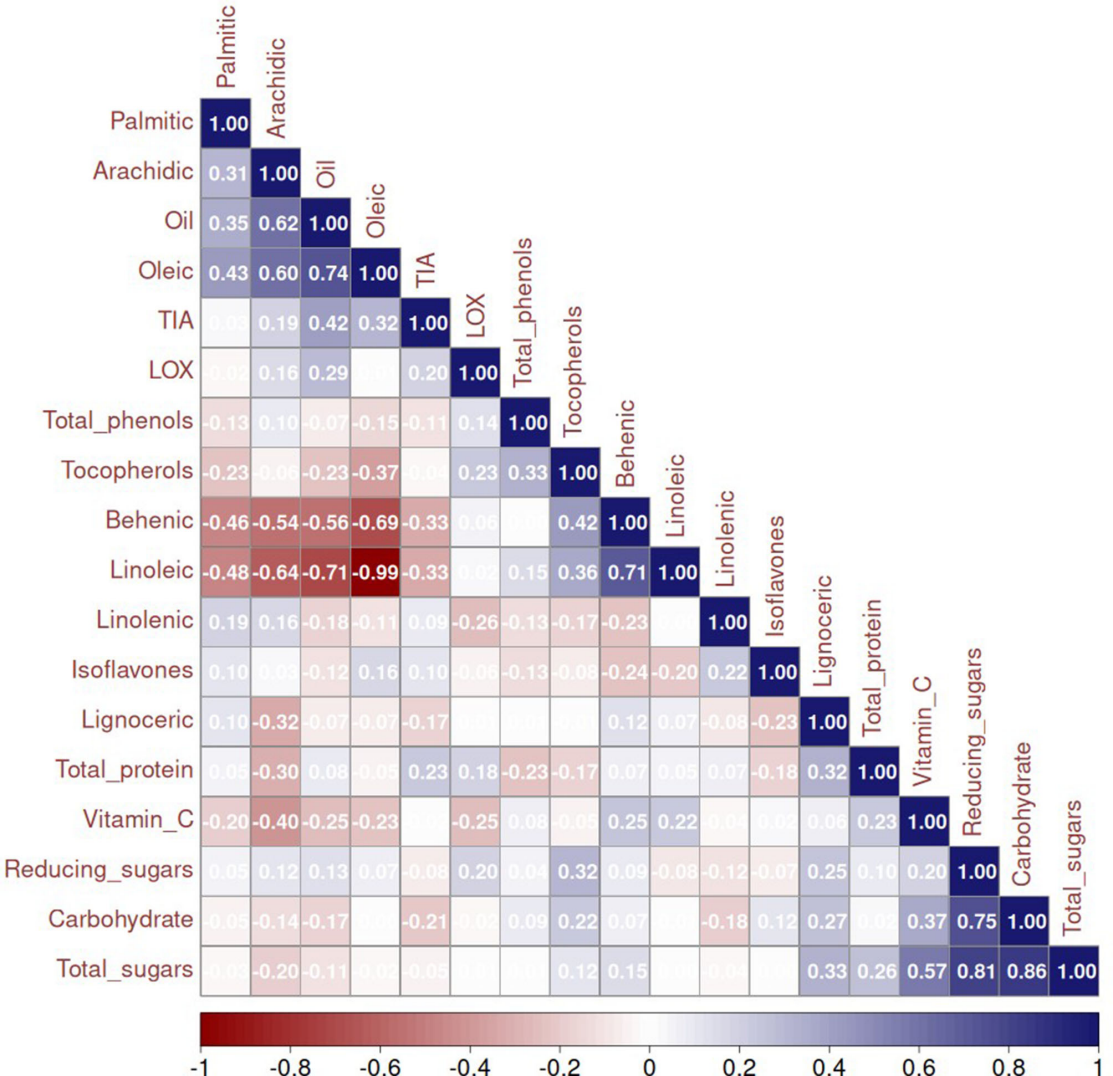
Correlation between all the parameters estimated (empty cells represent no correlation) TIU, trypsin inhibitor units.

The correlations among various parameters were studied to understand their interrelationships (Figure 3). Our analysis revealed a significantly positive correlation between total oil content and oleic acid. This finding is notable because high oleic acid content is a desirable trait in oilseed crops, enhancing the oil's oxidative stability, which is beneficial for cooking and industrial uses. The positive correlation suggests that breeding for higher oil content could also increase oleic acid levels, thereby improving the nutritional value and stability of the oil. Conversely, we found significant negative correlations between oleic acid and linoleic acid, as well as between total oil content and linoleic acid. This is expected considering the substrate—product relationship, in which oleic acid undergoes desaturation to form linoleic acid. Linoleic acid, a polyunsaturated fatty acid, is essential in human diets, but excessive amounts can decrease oxidative stability in oils. This finding is crucial for developing oilseed varieties with high oil content and enhanced stability, as reducing linoleic acid while increasing oleic acid can improve the oil's shelf life and health benefits. Moreover, lipoxygenase acts on the linoleic acid to generate off-flavor-causing compounds. Our research aligns with previous findings by Kumar et al. (24). No significant positive or negative correlation of isoflavones, tocopherols, and linolenic acid with any other parameters, as shown in Figure 3, may be due to the distinct genetic and biochemical pathways governing the synthesis of these compounds compared to those controlling oil content and major fatty acid composition.

**Figure 4:**
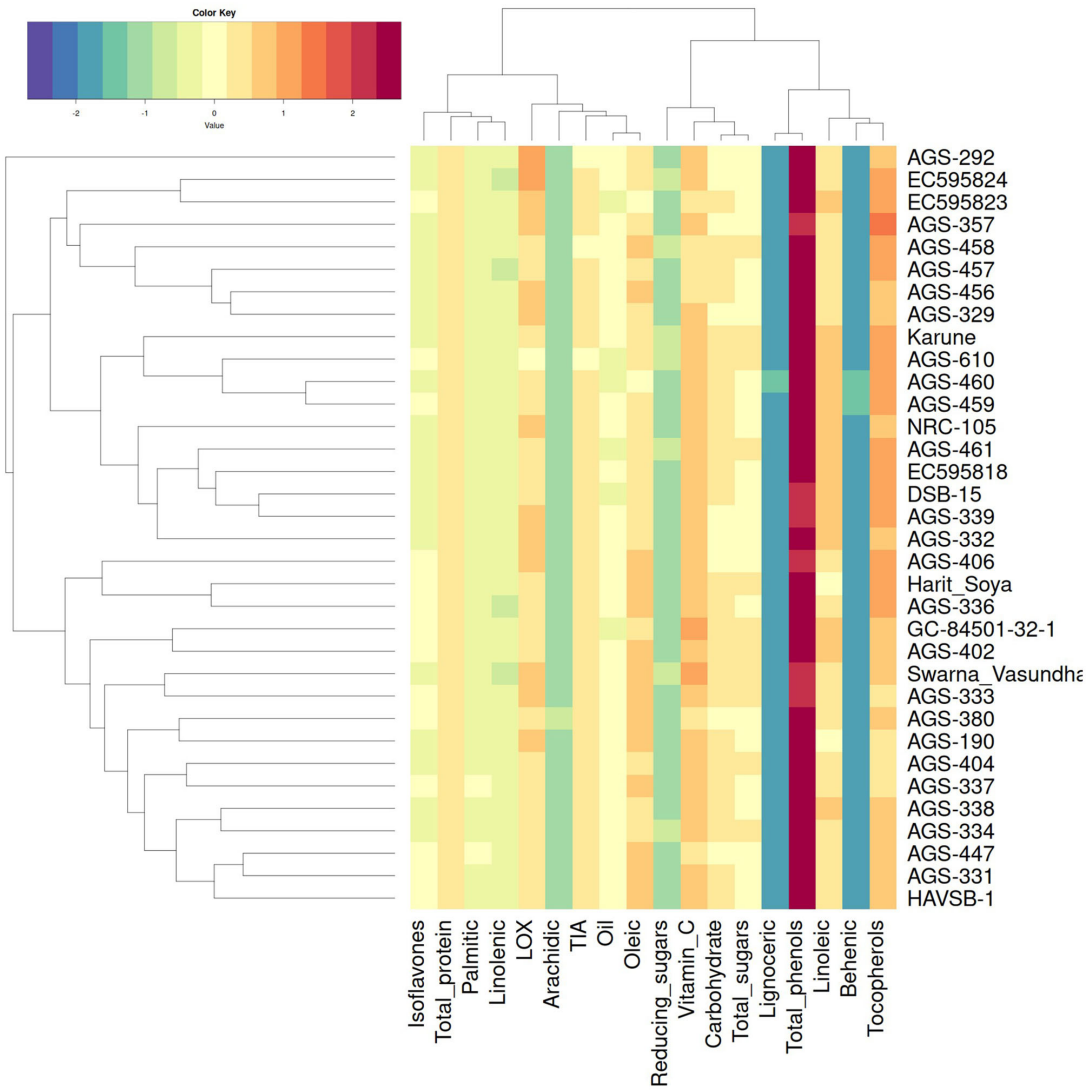
Heat map with hierarchical clustering of genotypes and parameters. The color gradient signifies standardized *Z*-scores of biochemical parameters across genotypes, quantifying the relative deviation of each value from the overall mean.

The hierarchical cluster analysis generated a heatmap that illustrates genotype-parameter associations. Genotypes were categorized into two main groups, Groups A and B, with several subgroups within each. Group A included genotypes such as AGS-406, Harit Soya, AGS-336, GC-84501-32-1, AGS-402, Swarna Vasundhara, AGS-333, AGS-380, AGS-190, AGS-404, AGS-337, AGS-338, AGS-334, AGS-447, AGS-331, and HAVSP-1, while the remaining genotypes were placed in Group B. Interestingly, AGS-292 remained as an outlier, suggesting the unique genetic attributes. This could be due to its fatty acid composition, which is different from other genotypes. Similarly, parameters were clustered into Group I and Group II, as shown in Figure 4. The majority of nutritional components are strongly associated with genotypes present in Group A. Bioactive compounds such as tocopherols and isoflavones are highly concentrated in the genotypes categorized in Group B. This clustering pattern provides insights into breeding programs, facilitating the enhancement of specific desirable traits. This detailed clustering pattern aids in strategic genotype selection and breeding for optimal trait enhancement that can help combat malnutrition more effectively.

**Figure 5:**
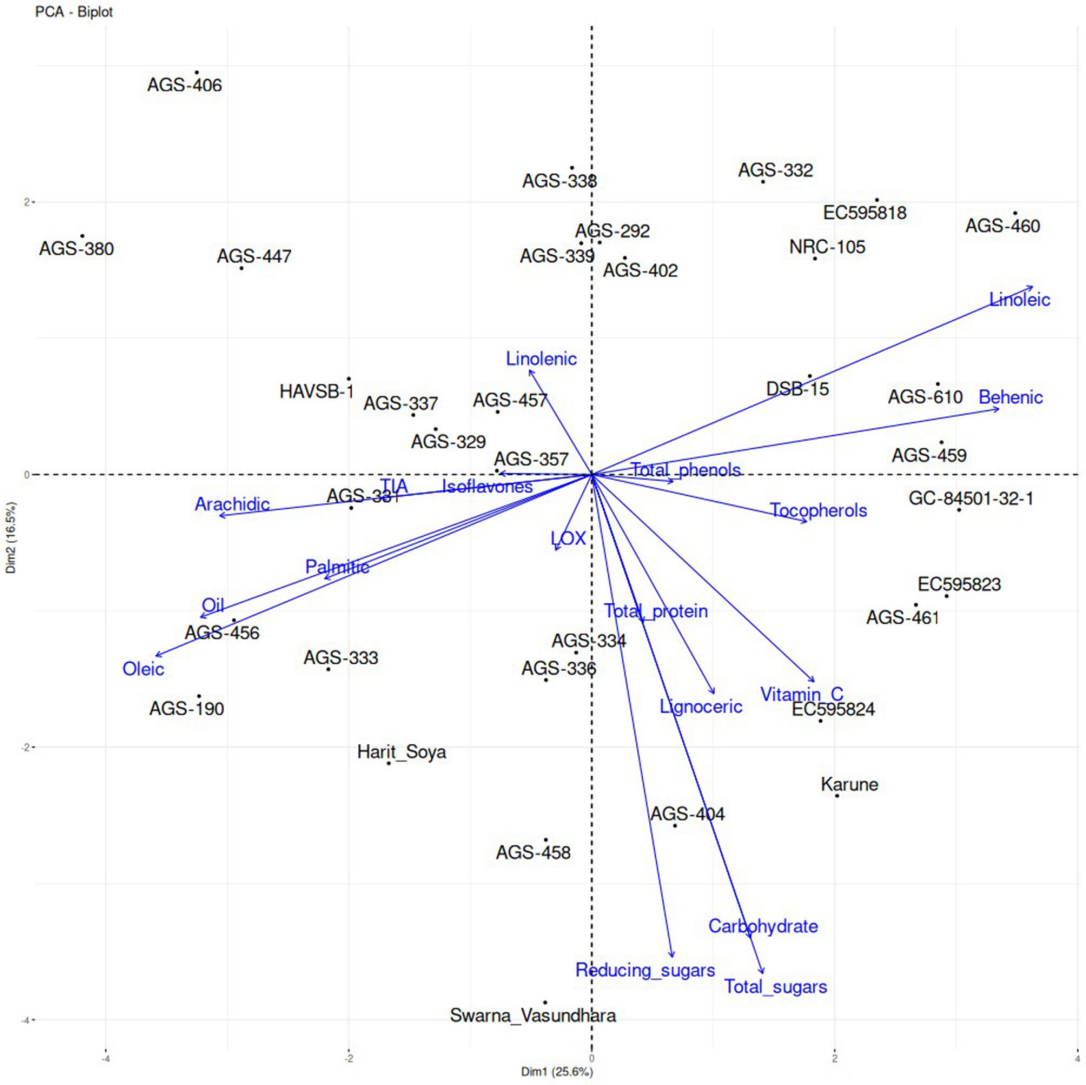
Principal component analysis (PCA) of vegetable soybean genotypes with various biochemical parameters under study.

The principal component analysis (PCA) of the genotypes revealed that the first principal component (PC1) accounted for 25.6% of the total variation, while the second principal component (PC2) explained 16.5%, indicating that much of the variability within the dataset could be attributed to PC1, with a significant, although smaller, contribution from PC2. The genotypes were scattered across the graph with no apparent clustering, suggesting a wide range of genetic variation and biochemical profiles. Oil content and oleic acid were strongly positively correlated with both principal components, indicating that these traits are major contributors to the observed variability. In contrast, linoleic acid showed a negative correlation with both components, positioned on the same line but in the opposite direction to oleic acid, suggesting that as oleic acid increases, linoleic acid decreases. Genotypes such as AGS-190 and AGS-456 are positioned near these variables, indicating a high concentration of oil content and oleic acid in these genotypes. Similarly, the trends of other parameters and genotypes showing higher concentrations of specific traits are depicted in Figure 5. The total protein content, carbohydrates, and total sugars aligned on the same vector in the PCA biplot, indicating a very strong positive correlation among these parameters. Additionally, reduced sugars are closely aligned with this vector, suggesting a significant association. This strong interdependence highlights a shared underlying biochemical or metabolic pathway, providing valuable insights into the functional relationship between these components. These insights are pivotal for developing targeted breeding strategies to optimize both the nutritional and industrial value of the crop. By utilizing these PCA findings, breeders can strategically choose and breed genotypes that align with specific breeding goals, thereby enhancing crop quality and associated health benefits. For example, targeting traits, such as oleic acid and linoleic acid, through selective breeding can lead to the optimization of the oil quality of vegetable soybean genotypes.

### 3.5 Superior genotypes with multiple desirable traits

A two-way ANOVA was conducted, and the resulting high *F* values across all traits indicate substantial variation caused by genotypes, while the extremely low *P*-values (< 0.0001) confirm the statistical significance of these effects (Table 2). This finding suggests that genotypes play a dominant role in determining these traits, highlighting meaningful differences among individual genotypes. This study identified several genotypes with multiple desirable traits, which could be valuable for further breeding efforts. A detailed list of such genotypes with multiple desirable traits is listed in Table 4. For instance, AGS-336 not only exhibited high isoflavone content but also had high oleic acid and low linoleic acid content, contributing to oil stability without compromising tocopherols. Similar is the case with AGS-406 and EC595823. The genotype AGS-292 had low anti-nutrient factors, trypsin inhibitors, along with low levels of lignoceric and linolenic acids, indicating potential for improved consumption. AGS-190 is a promising genotype for oil extraction and oxidative stability of oil owing to its high oil content, high oleic acid, and low linolenic acid. Similar results were found in AGS-357, AGS-456, and AGS-337. These genotypes aim to address malnutrition by providing improved nutritional profiles and reduced anti-nutrient factors. This combination of health-promoting fatty acids, antioxidants, and improved nutrient absorption creates a holistic approach to health, offering benefits across multiple systems, from cardiovascular to metabolic health.

**Table 4:** Elite vegetable soybean genotypes with multiple desirable traits.

**Cultivar**	**Additional traits**
**High isoflavones (**>**20 mg/100 g) for promoting general health**	
AGS-336	High tocopherols, high oleic acid, low linoleic acid
AGS-406	High oleic acid, low saturated fats, low linoleic acid
**High tocopherols (**>**120 mg/100 g) for antioxidants**	
**and promoting cardiovascular health**	
AGS-357	High oleic acid, low linoleic acid
AGS-460	Low saturated fats, high linoleic acid
**Low trypsin inhibitor activity (**<**20 TIU/mg) for better**	
**nutrient availability**	
AGS-292	Low lignoceric and linolenic acids
AGS-610	High polyunsaturated fatty acids (PUFAs)
**High oil content (**>**18%) for high-calorie food**	
AGS-190	High oleic acid, low linoleic acid
AGS-456	High oleic acid, low linoleic acid
**High oleic acid (**>**48%)**	
AGS-190	High oil content, low linoleic acid
HAVSB-1	Low PUFAs
**High linoleic acid (**>**50%)**	
AGS-460	High tocopherol, low saturated fats
EC595823	High isoflavones, high tocopherols, low saturated fats
**Low linoleic acid (**<**25%)**	
AGS-190	High oil content, high oleic acid, low behenic acid
AGS-337	High oleic acid, low arachidic acid

## Data Availability

The original contributions presented in the study are included in the article/Supplementary material, further inquiries can be directed to the corresponding authors.

## References

[1] Kapoor R Singh N Nguyen PH Singh SK Dwivedi LK Pedgaonkar S. How is India Doing on Malnutrition and Non-Communicable Diseases? Insights from the National Family Health Surveys 2005-06 to 2019-21. Washington, DC: International Food Policy Research Institute (2022). 10.2499/p15738coll2.136565

[2] Li X Siddique KHM. Future smart food: harnessing the potential of neglected and underutilized species for Zero Hunger. Matern Child Nutr. (2020) 16(Suppl 3):e13008. 10.1111/mcn.13008 33347726 PMC7752121

[3] Qin P Wang T Luo Y. A review on plant-based proteins from soybean: Health benefits and soy product development. J Agr Food Res. (2022) 7:100265. 10.1016/j.jafr.2021.100265

[4] Sahin I Bilir B Ali S Sahin K Kucuk O. Soy isoflavones in integrative oncology: increased efficacy and decreased toxicity of cancer therapy. Integr Cancer Ther. (2019) 18:1534735419835310. 10.1177/1534735419835310 30897972 PMC6431760

[5] Fan Y Wang M Li Z Jiang H Shi J Shi X. Intake of soy, soy isoflavones and soy protein and risk of cancer incidence and mortality. Front Nutr. (2022) 9:847421. 10.3389/fnut.2022.847421 35308286 PMC8931954

[6] Konovsky J Lumpkin TA McClary D. Edamame: the vegetable soybean. In:O'Rourke AD, editor. Understanding the Japanese Food and Agrimarket: A Multifaceted Opportunity. CRC Press (1994). p. 173–81. 10.1201/9781003075172-15

[7] Nair RM Boddepalli VN Yan M-R Kumar V Gill B Pan RS. Global status of vegetable soybean. Plants. (2023) 12:609. 10.3390/plants12030609 36771696 PMC9920938

[8] Patil V Meti S Mansur CP Rajashekhara E Prabhaker I Hadimani HP. Kaviraja H. Nutritional studies on vegetable soybean [*Glycine max* (L) merrill], in northern dryzone of Karnataka, India. Int J Curr Microbiol App Sci. (2017) 6:5364–74. 10.20546/ijcmas.2017.612.501

[9] Agyenim-Boateng KG Zhang S Zhang S Khattak AN Shaibu A Abdelghany AM Qi J. The nutritional composition of the vegetable soybean (maodou) and its potential in combatting malnutrition. Front Nutr. (2022) 9:1034115. 10.3389/fnut.2022.1034115 36687682 PMC9849953

[10] Keatinge JDH Easdown WJ Yang RY Chadha ML Shanmugasundaram S. Overcoming chronic malnutrition in a future warming world: the key importance of mungbean and vegetable soybean. Euphytica. (2011) 180:129–41. 10.1007/s10681-011-0401-6

[11] Dubois M Gilles K Hamilton JK Rebers PA Smith F. A colorimetric method for the determination of sugars. Nature. (1951) 168:167. 10.1038/168167a0 14875032

[12] Chaudhari HA Mahatma MK Antala V Radadiya N Ukani P Tomar RS. Ethrel-induced release of fresh seed dormancy causes remodelling of amylase activity, proteomics, phytohormone and fatty acid profile of groundnut (*Arachis hypogaea* L). Physiol Mol Biol Plants. (2023) 29:829–42. 10.1007/s12298-023-01332-6 37520814 PMC10382464

[13] Standal BR. Nutritional value of proteins of oriental soybean foods. J Nutr. (1963) 81:279–85. 10.1093/jn/81.3.279 14083244

[14] Plaza L de Ancos B. Cano PM. Nutritional and health-related compounds in sprouts and seeds of soybean (*Glycine max*), wheat (*Triticum aestivum* L) and alfalfa (*Medicago sativa*) treated by a new drying method. Eur Food Res Technol. (2003) 216:138–44. 10.1007/s00217-002-0640-9

[15] Tewari K Kumar V Kumar A Bansal N Vinutha T Ali K. Molecular cloning and functional analysis of the promoter of γ-Tocopherol Methyl Transferase (γ-TMT) gene of soybean (*Glycine max*). 3 Biotech. (2018) 8:325. 10.1007/s13205-018-1347-3 30034989 PMC6050176

[16] Tewari K Kumari S Vinutha T Singh B Dahuja A. Gamma irradiation induces reduction in the off-flavour generation in soybean through enhancement of its antioxidant potential. J Radioanal Nucl Chem. (2014) 303:2041–51. 10.1007/s10967-014-3803-9

[17] Singleton VL Orthofer R Lamuela-Raventós RM. [14] Analysis of total phenols and other oxidation substrates and antioxidants by means of folin-ciocalteu reagent. In: *Oxidants and Antioxidants Part A Methods in Enzymology*. Elsevier (1999). p. 152–78. 10.1016/S0076-6879(99)99017-1

[18] Dhaubhadel S McGarvey BD Williams R Gijzen M. Isoflavonoid biosynthesis and accumulation in developing soybean seeds. Plant Mol Biol. (2003) 53:733–43. 10.1023/B:PLAN.0000023666.30358.ae 15082922

[19] Dahuja A Madaan TR. Estimation of parameters responsible for the generation of off-flavor in some Indian varieties of soybeans. Plant Foods Hum Nutr. (2003) 58:1–8. 10.1023/B:QUAL.0000041148.77475.fd12859008

[20] Liu K. Soybean trypsin inhibitor assay: further improvement of the standard method approved and reapproved by American oil chemists' society and American association of cereal chemists international. J Am Oil Chem Soc. (2019) 96:635–45. 10.1002/aocs.12205

[21] Mohamed AI Mebrahtu T. Rangappa M. Nutrient composition and anti-nutritional factors in selected vegetable soybean (*Glycine max* [L] Merr). Plant Foods Hum Nutr. (1991) 41:89–100. 10.1007/BF02196385 2017430

[22] Kumar V Rani A Goyal L Pratap D Billore SD Chauhan GS. Evaluation of vegetable-type soybean for sucrose, taste-related amino acids, and isoflavones contents. Int J Food Prop. (2011) 14:1142–51. 10.1080/10942911003592761

[23] Kumar V Rani A Rawal R. First Indian soybean variety free from off-flavour generating lipoxygenase-2 gene identified for release for commercial cultivation. Natl Acad Sci Lett. (2021) 44:477–80. 10.1007/s40009-021-01046-x

[24] Kumar V Rani A Dixit AK Pratap D Bhatnagar D. A comparative assessment of total phenolic content, ferric reducing-anti-oxidative power, free radical-scavenging activity, vitamin C and isoflavones content in soybean with varying seed coat colour. Food Res Int. (2010) 43:323–8. 10.1016/j.foodres.2009.10.019

[25] Rani A Kumar V Verma SK Shakya AK Chauhan GS. Tocopherol content and profile of soybean: genotypic variability and correlation studies. J Am Oil Chem Soc. (2007) 84:377–83. 10.1007/s11746-007-1040-x

[26] Zhang C Zhang Y Liu G Li W Xia S Li H. Effects of soybean protein isolates and peptides on the growth and metabolism of *Lactobacillus rhamnosus*. *J Funct Foods*. (2021) 77:104335. 10.1016/j.jff.2020.10433530934634

[27] Cabanos C Matsuoka Y Maruyama N. Soybean proteins/peptides: a review on their importance, biosynthesis, vacuolar sorting, and accumulation in seeds. Peptides. (2021) 143:170598. 10.1016/j.peptides.2021.170598 34153351

[28] Gillman JD Kim W-S Krishnan HB. Identification of a new soybean kunitz trypsin inhibitor mutation and its effect on bowman-birk protease inhibitor content in soybean seed. J Agric Food Chem. (2015) 63:1352–9. 10.1021/jf505220p 25608918

[29] Xu Y Cartier A Kibet D Jordan K Hakala I Davis S. Physical and nutritional properties of edamame seeds as influenced by stage of development. Food Measure. (2016) 10:193–200. 10.1007/s11694-015-9293-9

[30] Ly S Park BE Shim SI Kim MC Moon JY Chung JI. Breeding a black soybean line with green cotyledon free from lectin, KTI, P34, lipoxygenase, and stachyose. Euphytica. (2024) 220:131. 10.1007/s10681-024-03391-6

[31] Mohamed AI Rangappa M. Screening soybean (grain and vegetable) genotypes for nutrients and anti-nutritional factors. Plant Foods Hum Nutr. (1992) 42:87–96. 10.1007/BF02196075 1546056

[32] Doseděl M Jirkovský E Macáková K Krčmová LK Javorská L Pourová J. Vitamin C-sources, physiological role, kinetics, deficiency, use, toxicity, and determination. Nutrients. (2021) 13:615. 10.3390/nu13020615 33668681 PMC7918462

[33] Castoldi R Charlo HC de O Vargas PF Braz LT Carrão-Panizzi MC. Agronomic characteristics, isoflavone content and Kunitz trypsin inhibitor of vegetable soybean genotypes. Hortic Bras. (2011) 29:222–7. 10.1590/S0102-05362011000200015

[34] Kumar V Rani A Goyal L Vaishnav J Pratap D Dixit AK. Assessment of antioxidant constituents and anti-oxidative properties of vegetable soybean. Int J Food Prop. (2014) 17:536–44. 10.1080/10942912.2012.654559

[35] Ali E Hussain S Hussain N Kakar KU Shah JM Zaidi SHR. Tocopherol as plant protector: an overview of Tocopherol biosynthesis enzymes and their role as antioxidant and signaling molecules. Acta Physiol Plant. (2022) 44:20. 10.1007/s11738-021-03350-x

[36] Kumar V Rani A Dixit AK Bhatnagar D Chauhan GS. Relative changes in tocopherols, isoflavones, total phenolic content, and antioxidative activity in soybean seeds at different reproductive stages. J Agric Food Chem. (2009) 57:2705–10. 10.1021/jf803122a 19256542

[37] Zhao Y Xie C Wang P Gu Z Yang R. GABA regulates phenolics accumulation in soybean sprouts under NaCl stress. Antioxidants. (2021) 10:990. 10.3390/antiox10060990 34205788 PMC8235516

[38] Pabich M Marciniak B Kontek R. Phenolic compound composition and biological activities of fractionated soybean pod extract. Appl Sci. (2021) 11:10233. 10.3390/app112110233

[39] Aboushanab SA Khedr SM Gette IF Danilova IG Kolberg NA Ravishankar GA. Isoflavones derived from plant raw materials: bioavailability, anti-cancer, anti-aging potentials, and microbiome modulation. Crit Rev Food Sci Nutr. (2023) 63:261–87. 10.1080/10408398.2021.1946006 34251921

[40] Kumar S Banerjee S Kaur A Sasi M Kumari S Sachdev A. Isoflavones have a potential role in off-flavour scavenging, with IFS2 as a key in isoflavone accumulation in soybean seeds. Food Technol Biotechnol. (2023) 61:514–22. 10.17113/ftb.61.04.23.8231 38205057 PMC10775790

[41] Vicaş SI Chedea VS Socaciu C. Inhibitory effects of isoflavones on soybean lipoxygenase-1 activity. J Food Biochem. (2011) 35:613–27. 10.1111/j.1745-4514.2010.00405.x

