# Supplementary material for: Nutritional analysis of vegetable soybean [Glycine max (L.) Merrill] accessions in Eastern India
Source: Front Nutr. 2025 Sep 4;12:1643470. doi: 10.3389/fnut.2025.1643470 (PMC12443715; doi:10.3389/fnut.2025.1643470)
Supplement: Supplementary file 1 [file Table_1.docx]

Supplementary Material

Nutritional analysis of Vegetable Soybean [Glycine max (L.) Merrill] accessions in Eastern India

**Table S1: Mean performance of 34 vegetable soybean genotypes for green pod yield and related horticultural traits (c).**

| **SL. NO.** | **Genotypes** | **100-green seed weight of 2-seeded pods (g)** | **100-green seed weight of 3-seeded pods (g)** | **Graded green pod yield per plant (kg)** | **Green  seed yield per plant (kg)** |
| --- | --- | --- | --- | --- | --- |
| 1 | HAVSB-1 | 50.00 | 51.67 | 0.273 | 0.137 |
| 2 | AGS-190 | 49.67 | 55.67 | 0.291 | 0.142 |
| 3 | AGS-331 | 41.33 | 45.67 | 0.281 | 0.132 |
| 4 | AGS-332 | 43.33 | 47.33 | 0.161 | 0.120 |
| 5 | AGS-333 | 48.33 | 50.00 | 0.273 | 0.145 |
| 6 | AGS-334 | 42.67 | 46.00 | 0.238 | 0.150 |
| 7 | AGS-336 | 46.11 | 50.00 | 0.256 | 0.130 |
| 8 | AGS-337 | 50.00 | 52.00 | 0.207 | 0.163 |
| 9 | AGS-338 | 50.00 | 55.33 | 0.223 | 0.122 |
| 10 | AGS-292 | 40.33 | 43.33 | 0.264 | 0.162 |
| 11 | AGS-329 | 68.67 | 73.33 | 0.283 | 0.159 |
| 12 | AGS-339 | 72.00 | 74.00 | 0.245 | 0.152 |
| 13 | AGS-357 | 76.67 | 79.33 | 0.283 | 0.162 |
| 14 | AGS-380 | 71.33 | 72.67 | 0.276 | 0.167 |
| 15 | AGS-402 | 72.22 | 74.00 | 0.267 | 0.132 |
| 16 | AGS-404 | 53.00 | 55.33 | 0.379 | 0.171 |
| 17 | AGS-406 | 73.33 | 76.67 | 0.233 | 0.129 |
| 18 | AGS-447 | 76.00 | 79.33 | 0.312 | 0.173 |
| 19 | AGS-456 | 53.33 | 59.00 | 0.316 | 0.177 |
| 20 | AGS-457 | 77.67 | 78.00 | 0.306 | 0.171 |
| 21 | AGS-458 | 68.00 | 73.33 | 0.399 | 0.217 |
| 22 | AGS-459 | 71.50 | 76.00 | 0.278 | 0.190 |
| 23 | AGS-460 | 63.33 | 66.00 | 0.215 | 0.133 |
| 24 | AGS-461 | 70.00 | 73.33 | 0.219 | 0.130 |
| 25 | AGS-610 | 75.56 | 77.00 | 0.277 | 0.170 |
| 26 | GC-84501-32-1 | 55.56 | 61.43 | 0.363 | 0.181 |
| 27 | Harit Soya | 42.00 | 51.33 | 0.242 | 0.120 |
| 28 | EC595818 | 70.00 | 75.33 | 0.255 | 0.150 |
| 29 | EC595823 | 72.17 | 74.00 | 0.288 | 0.163 |
| 30 | EC595824 | 72.67 | 82.33 | 0.284 | 0.172 |
| 31 | DSB-15 | 52.00 | 56.00 | 0.188 | 0.123 |
| 32 | NRC-105 | 75.56 | 77.00 | 0.251 | 0.153 |
| 33 | Karune | 68.13 | 72.00 | 0.293 | 0.182 |
| 34 | Swarna Vasundhara | 50.67 | 52.00 | 0.289 | 0.150 |
|  | **SE (m) ±** | **1.59** | **1.71** | **0.007** | **0.008** |
|  | **CV (%)** | **4.54** | **4.61** | **4.77** | **8.84** |
|  | **CD 0.01** | **5.97** | **6.41** | **0.028** | **0.029** |
|  | **CD 0.05** | **4.51** | **4.82** | **0.021** | **0.022** |

**Table S2: Mean performance of Vegetable Soybean genotypes for fatty acids (%)**

| **Genotype** | **Palmitic acid** | **Oleic acid** | **Linoleic acid** | **Linolenic acid** | **Arachidic acid** | **Behemic acid** | **Lignoceric** |
| --- | --- | --- | --- | --- | --- | --- | --- |
| HAVSB-1 | 12.11 ± 0.24^a-g^ | 48.92 ± 0.64^k^ | 27.97 ± 0.20^de^ | 7.47 ± 0.27^abc^ | 3.23 ± 0.16^e-i^ | 0.31±0.04^de^ | 0.28 ± 0.01^a-c^ |
| AGS-190 | 12.87 ± 0.19^a-e^ | 52.10 ± 0.14^l^ | 23.16 ± 0.40^a^ | 7.94 ± 0.04^a-h^ | 3.65 ± 0.10^ij^ | 0.21±0.14^bc^ | 0.33 ± 0.00^b-d^ |
| AGS-331 | 12.92 ± 0.33^a-d^ | 46.58 ± 0.26^j^ | 29.07 ± 0.22^ef^ | 8.49 ± 0.10^d-i^ | 2.58 ± 0.10^a-e^ | 0.23±0.11^c^ | 0.46 ± 0.00^e^ |
| AGS-332 | 11.26 ± 0.22^g^ | 27.62 ± 0.22^c^ | 50.55 ± 0.46^no^ | 7.42 ± 0.22^ab^ | 2.70 ± 0.10^a-f^ | 0.43±0.09^i-k^ | 0.33 ± 0.02^b-d^ |
| AGS-333 | 13.09 ± 0.43^ab^ | 48.73 ± 0.25^k^ | 26.50 ± 0.11^cd^ | 8.53 ± 0.06^e-i^ | 2.89 ± 0.06^b-h^ | 0.35±0.01^d-g^ | 0.32 ± 0.00^b-d^ |
| AGS-334 | 11.86 ± 0.20^c-g^ | 37.04 ± 0.28^f^ | 34.94 ± 0.36^h^ | 9.54 ± 0.24^k-mn^ | 3.06 ± 0.07^d-i^ | 0.34±0.00^d-g^ | 0.35 ± 0.00^cd^ |
| AGS-336 | 12.18 ± 0.13^a-g^ | 46.80 ± 0.29^j^ | 28.07 ± 0.24^de^ | 7.55 ± 0.22^abc^ | 2.42 ± 0.16^a-d^ | 0.30±0.04^de^ | 0.34 ± 0.01^cd^ |
| AGS-337 | 12.85 ± 0.14^a-e^ | 48.79 ± 0.23^k^ | 24.83 ± 0.26^b^ | 8.12 ± 0.11^b-i^ | 2.63 ± 0.26^a-f^ | 0.30±0.04^de^ | 0.35 ± 0.02^cd^ |
| AGS-338 | 11.96 ± 0.20^a-g^ | 33.84 ± 0.24^e^ | 40.77 ± 0.30^j^ | 10.13 ± 0.17^n^ | 2.78 ± 0.09^a-g^ | 0.33±0.01^d-g^ | 0.28 ± 0.01^a-c^ |
| Swarna Vasundhara | 12.32 ± 0.29^a-g^ | 48.61 ± 0.18^k^ | 28.47 ± 0.29^ef^ | 7.65 ± 0.20^a-d^ | 2.75 ± 0.13^a-g^ | 0.34±0.00^d-g^ | 0.34 ± 0.01^cd^ |
| AGS-292 | 12.35 ± 0.24^a-g^ | 37.50 ± 0.33^fg^ | 39.18 ± 0.15^i^ | 7.58 ± 0.19^a-c^ | 2.91 ± 0.04^c-h^ | 0.45±0.10^jk^ | 0.36 ± 0.04^cd^ |
| AGS-329 | 11.92 ± 0.08^b-g^ | 43.29 ± 0.49^i^ | 33.36 ± 0.59^gh^ | 8.33 ± 0.11^c-i^ | 3.60 ± 0.20^ij^ | 0.36±0.01^e-h^ | 0.35 ± 0.02^cd^ |
| AGS-339 | 12.71 ± 0.20^a-f^ | 30.65 ± 0.52^d^ | 46.02 ± 0.33^k^ | 7.81 ± 0.21^a-g^ | 2.51 ± 0.22^a-d^ | 0.40±0.05^g-j^ | 0.30 ± 0.01^a-d^ |
| AGS-357 | 12.21 ± 0.17^a-g^ | 43.07 ± 0.42^hi^ | 33.09 ± 0.11^g^ | 8.13 ± 0.07^b-i^ | 3.32 ± 0.13f-i | 0.37±0.03^f-i^ | 0.31 ± 0.00^a-d^ |
| AGS-380 | 13.04 ± 0.27^a-c^ | 43.59 ± 0.27^i^ | 29.32 ± 0.51^ef^ | 9.77 ± 0.12^n^ | 4.14 ± 0.01^j^ | 0.12±0.23^a^ | 0.23 ± 0.01^a^ |
| AGS-402 | 11.60 ± 0.13^fg^ | 39.13 ± 0.12^g^ | 38.84 ± 0.43^i^ | 8.22 ± 0.06^b-i^ | 2.15 ± 0.11^a^ | 0.39±0.05^g-j^ | 0.28 ± 0.01^a-c^ |
| AGS-404 | 12.46 ± 0.08^a-g^ | 37.90 ± 0.44^fg^ | 37.85 ± 0.51^i^ | 8.74 ± 0.11^h-k^ | 2.35 ± 0.13^a-d^ | 0.18±0.17^ab^ | 0.47 ± 0.01^e^ |
| AGS-406 | 12.41 ± 0.19^a-g^ | 41.47 ± 0.47^h^ | 32.81 ± 0.24^g^ | 8.91 ± 0.07^i-m^ | 3.41 ± 0.24^g-j^ | 0.14±0.21^a^ | 0.24 ± 0.00^ab^ |
| AGS-447 | 13.15 ± 0.21^a^ | 45.51 ± 0.34^j^ | 30.01 ± 0.20^f^ | 8.23 ± 0.20^b-i^ | 2.92 ± 0.07^c-h^ | 0.18±0.17^ab^ | 0.34 ± 0.01^cd^ |
| AGS-456 | 13.11 ± 0.35^ab^ | 45.48 ± 0.28^j^ | 29.41 ± 0.33^ef^ | 7.93 ± 0.06^a-h^ | 3.30 ± 0.09^f-i^ | 0.16±0.19^ab^ | 0.36 ± 0.00^cd^ |
| AGS-457 | 11.96 ± 0.04^a-g^ | 41.51 ± 0.78^h^ | 34.38 ± 0.57^gh^ | 7.24 ± 0.17^a^ | 3.24 ± 0.16^e-i^ | 0.39±0.05^g-j^ | 0.38 ± 0.00^c-e^ |
| AGS-458 | 12.21 ± 0.27^a-g^ | 45.94 ± 0.34^j^ | 29.34 ± 0.16^ef^ | 7.71 ± 0.30^a-f^ | 3.44 ± 0.27^g-j^ | 0.32±0.02^d-f^ | 0.35 ± 0.01^cd^ |
| AGS-459 | 11.75 ± 0.11^d-g^ | 26.18 ± 0.51^a-c^ | 48.97 ± 0.36^l-n^ | 8.54 ± 0.21^e-i^ | 2.60 ± 0.09^a-e^ | 0.42±0.07^h-k^ | 0.35 ± 0.02^cd^ |
| AGS-460 | 11.84 ± 0.24^c-g^ | 24.96 ± 0.07^a^ | 51.76 ± 0.23^o^ | 7.94 ± 0.26^a-h^ | 2.18 ± 0.09^ab^ | 0.45±0.10^jk^ | 0.29 ± 0.01^a-c^ |
| AGS-461 | 12.90 ± 0.18^a-d^ | 25.97 ± 0.39^a-c^ | 49.45 ± 0.25^l-n^ | 8.63 ± 0.12^g-j^ | 2.44 ± 0.11^a-d^ | 0.43±0.08^i-k^ | 0.33 ± 0.01^b-d^ |
| AGS-610 | 12.43 ± 0.19^a-g^ | 26.82 ± 0.24^bc^ | 48.26 ± 0.52^lm^ | 8.95 ± 0.09^i-m^ | 2.63 ± 0.19^a-f^ | 0.48±0.13^kl^ | 0.37 ± 0.01^c-e^ |
| GC-84501-32-1 | 11.68 ± 0.24^e-g^ | 30.81 ± 0.30^d^ | 45.56 ± 0.21^k^ | 7.54 ± 0.21^a-c^ | 2.36 ± 0.04^a-d^ | 0.42±0.08^i-k^ | 0.32 ± 0.00^a-d^ |
| Harit Soya | 12.46 ± 0.23^a-g^ | 45.89 ± 0.27^j^ | 25.06 ± 0.47^bc^ | 9.41 ± 0.05^j-n^ | 3.50 ± 0.04^h-j^ | 0.29±0.06^d^ | 0.35 ± 0.01^cd^ |
| EC595818 | 11.78 ± 0.21^d-g^ | 27.01 ± 0.07^bc^ | 48.31 ± 0.58^lm^ | 8.53 ± 0.11^e-i^ | 2.47 ± 0.06^a-d^ | 0.50±0.16^l^ | 0.39 ± 0.01^de^ |
| EC595823 | 12.49 ± 0.32^a-g^ | 25.30 ± 0.66^ab^ | 50.17 ± 0.44^no^ | 8.57 ± 0.12^f-i^ | 2.32 ± 0.06^a-c^ | 0.44±0.10^jk^ | 0.40 ± 0.07^de^ |
| EC595824 | 11.59 ± 0.21^f-g^ | 31.13 ± 0.42^d^ | 45.38 ± 0.55^k^ | 7.55 ± 0.23^a-c^ | 2.62 ± 0.13^a-f^ | 0.43±0.08^i-k^ | 0.32 ± 0.01^b-d^ |
| DSB-15 | 12.92 ± 0.11^a-d^ | 27.37 ± 0.32^c^ | 48.00 ± 0.10^l^ | 8.63 ± 0.09^g-j^ | 2.54 ± 0.05^a-e^ | 0.53±0.19^l^ | 0.37 ± 0.00^c-e^ |
| NRC-105 | 11.43 ± 0.21^g^ | 27.28 ± 0.44^c^ | 49.87 ± 0.39^m-o^ | 8.75 ± 0.03^h-l^ | 2.24 ± 0.03^a-c^ | 0.35±0.00^d-g^ | 0.35 ± 0.04^cd^ |
| Karune | 12.25 ± 0.11^a-g^ | 30.73 ± 0.27^d^ | 45.41 ± 0.47^k^ | 7.70 ± 0.10^a-e^ | 2.80 ± 0.10^a-g^ | 0.38±0.04^f-j^ | 0.33 ± 0.01^b-d^ |
